# Leisure-related injuries in children and adolescents: a generational comparison of 20 years in a level 1 pediatric trauma center

**DOI:** 10.1186/s40621-026-00672-7

**Published:** 2026-03-28

**Authors:** Britta Chocholka, Iryna Yegorova, Antonia Schwarz, Lara Marie Bogensperger, Vanessa Groß, Stephan Payr, Manuela Jaindl

**Affiliations:** 1 University Clinic of Orthopedics and Trauma Surgery, Department of Trauma Surgery, Medical University of Vienna, Waehringer Guertel 18-20, Vienna, 1090 Austria; 2 Section of Pediatric Trauma Surgery, Department of Trauma Surgery, University Clinic of Orthopedics and Trauma Surgery, Medical University of Vienna, Vienna, Austria

**Keywords:** Injury rate, Children, Adolescents, Leisure-time, Generational patterns

## Abstract

**Background:**

Behavioral studies show changes and an overall decline in physical activity and general health literacy among children and adolescents. The objective of this study is to analyze and compare the incidence and distribution of leisure-time-related injuries among children and adolescents aged 0–17 years, examining two distinct time periods: 2000–2004 and 2015–2019.

**Methods:**

This retrospective analysis was conducted on a cohort of 27,761 children and adolescents who received treatment at our Level-1-Trauma Department due to leisure-time associated activities that occurred between 2000 and 2004 and 2015–2019. Data were stratified by age group, injury type, and anatomical region of injury.

**Results:**

The proportion of patients with leisure-related injuries among all injuries per period was significantly lower in the years 2015–2019 (total: 55,971 patients) than in the 2000–2004 period (total: 46,172 patients) (22%; 12,540/55,971 vs. 33%; 15,221/46,173; *p* < 0.001). Over the past twenty years, adolescents (11–17 years) revealed significantly fewer injuries (63.4%; 9651/15221 in 2000–2004 vs. 50.8%; 6367/12540 in 2015–2019; *p* < 0.001) while children up to the age of 5 represented an increasingly vulnerable age group (13%; 1975/15221 in 2000–2004 vs. 22.1%; 2771/12540 in 2015–2019; *p* < 0.001). In both time frames, majority of patients were male. Most common injuries were limb injuries, although age-dependent variations were observed.

**Conclusion:**

The findings reveal substantial shifts in pediatric injury patterns and a significant decrease of leisure time injuries in children and adolescents in the investigated time period of 20 years. These trends are indicative of changes in physical activity behaviors and evolving recreational preferences.

## Introduction

Injuries are recognized as a global public health problem among children and adolescents [1–3]. Numerous studies have been conducted to investigate injury-related morbidity and mortality, as well as injury patterns, contributing factors and potential improvements [4–6]. These insights led to significant changes in public health strategies over time, including enhanced traffic safety measures, sports regulations, and psychological support programs. Consequently, the number of fatal accidents decreased, as did the overall number of injuries among children and adolescents [6, 7]. However, the reduction in the number of injuries may also be due to changes in behavior and an overall decline in physical activity and general health awareness, rather than improved safety regulations alone [8–11]. At least one in five children aged six to nine years does not accumulate the amount of physical activity recommended by the WHO per day [12]. Similar rates are also reported for older children [13]. More recent studies have focused on the impact of the COVID-19 pandemic on children’s activity levels. These studies report alarmingly low activity levels in the pediatric population during and after the COVID-19 pandemic [14, 15]. However, this trend had already been apparent before [16–18]. This lower level of activity appears to be linked to an increase in health problems among children, such as obesity, metabolic syndrome, higher cardiometabolic risk and lower bone quality, which is important to avoid further deterioration in children’s health and negative impacts on the health system in general [10, 19–22]. Such a reduction in activity among the pediatric population may be accompanied by a decline in leisure and sports-related injuries. Furthermore, the activities and behaviors of young people are frequently confronted with new popular and changing trends (e-scooters, organized sports and contact sports) [23–25]. Although many epidemiological studies have examined the frequency of pediatric injuries, constant re-evaluation is necessary due to new emerging trends to track the current landscape of pediatric injuries [26]. Furthermore, little research addresses generational differences, temporal development, and shifts in injury patterns, and examines children and adolescent behavior from a trauma perspective. Therefore, this study aims to analyze and compare the frequency and distribution of leisure-time-related injuries among children and adolescents aged ≤17 years, examining two distinct time periods: 2000–2004 and 2015–2019. The study examines variables such as age groups, injury types, and anatomical regions affected in order to identify significant changes and trends in physical activity that have occurred over the 20-year period.

## Materials and methods

**Fig. 1:**
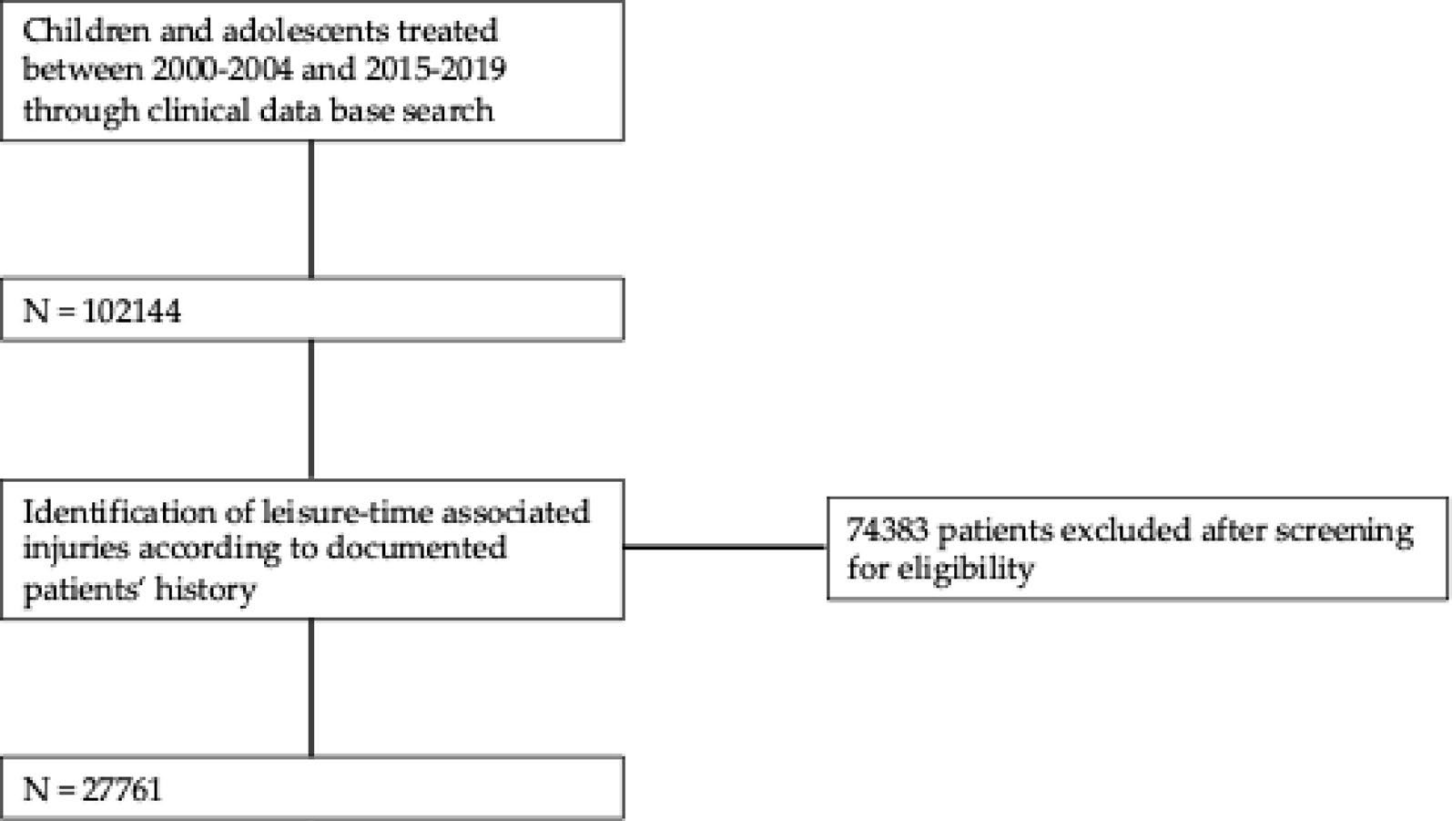
Process of identification of eligible study population

This retrospective single-center cohort study was conducted at the Department of Trauma Surgery at the Medical University of Vienna. The Ethics Committee of the Medical University of Vienna approved this study (EC-Code: 2269/2024) on 26 June 2025 in accordance with the latest version of the Declaration of Helsinki. All patients ≤17 years who were treated at the Department of Trauma Surgery at Vienna General Hospital/Medical University of Vienna between 2000 and 2004 and 2015–2019 due to a leisure-time injury were included. Data were retrieved from hospital records and screened for eligibility. Eligible participants were patients, who had sustained injuries during leisure-time activities (Fig. 1). Patients older 18 and older years of age or patients with injuries attributable to causes other than leisure activities or unknown mechanisms (e.g. injuries at work, at school, in motor vehicle accidents, etc.) were excluded. The demographic data of the entire cohort were available for analysis. In total, 238 patients (1.8%) had no recorded diagnosis, however contributed to the entire demography as such and were included in the overall statistical analysis. In instances where data pertaining to a specific parameter was not available, it was designated as ‘not recorded’. The rationale behind the exclusion of sports injuries sustained in educational institutions is due to the fact that all individuals are required to attend school and participate in sporting activities. The objective of the study was to observe alterations in leisure time and voluntary activities exclusively. By choosing pre-COVID time frames authors aimed to reduce any potential biases resulting from local regulations during the pandemic.

For each observation period, only the primary diagnosis was considered. Data were extracted retrospectively from electronic hospital records and screened to identify eligible cases based on the inclusion and exclusion criteria described above. Key information extracted included patients’ age, sex, primary injury diagnosis, anatomical region of injury, injury mechanism, and observation period (2000–2004 or 2015–2019). The data were stratified by age group (≤5 years, 6–10 years, 11–14 years, and 15–17 years), injury type, and anatomical region of injury to allow for subgroup analyses. The formation of age groups was based on the structure of the local school system. Injury type was coded based on the primary diagnosis recorded in the patient file. Subsequently, injuries were also categorized by severity. Severe injuries included fractures (ICD chapter S and T), injuries to internal organs (ICD chapter S 27 thorax, S 36 abdomen, S 37 pelvis), intracranial hematoma or oedema (ICD chapter I 62), traumatic amputations and wounds (ICD chapter S 00-T 98) in general. Moderate injuries included injuries to ligaments, muscle, tendons, soft tissue (ICD chapter S) and head contusions (ICD S00.95). Other injuries as contusions other than the head and strains (Chapter S and T14.05 for a contusion in an unspecified region of the body) were categorized as mild. Anatomical region of injury was classified into five categories (head, upper extremity, lower extremity, thorax/spine, and internal organs). For head injuries that were further categorized as contusion, concussion, fracture, intracranial hemorrhage. Data were entered into a standardized, anonymized database to ensure confidentiality.

### Statistical analysis

Descriptive data (mean ± standard deviation (SD)/percentages) were reported for the entire patient cohort. In order to provide an epidemiological overview, the parameters described above were included. For metric variables (age), mean values and standard deviation values and for categorial variables (region of injury, sex, injury mechanism), frequencies and percent-ages were determined and presented in absolute (abs.) numbers and relative (rel.%) percentages. Categorical data (injury region, injury type, age groups) were compared between the two observation periods (2000–2004 and 2015–2019) using Chi-squared Test. Initially, the data underwent an analysis to identify statistical differences on a year-by-year basis within each cohort. Subsequent to this, an analysis of the aggregate observation period was conducted. Bonferroni correction was applied to account for multiple comparisons. Statistical significance was set at a level of *p* < 0.05. Both the statistical analysis and the design of figures and tables were performed using Microsoft Excel (Version 16.90.2, Microsoft Corp., Redmond, WA, USA) and SPSS software (Version 29.0.2.0., IBM corp., Chicago, IL, USA).

## Results

### Demographics

In total, 27,761 eligible cases were included for analysis. Of the overall study population (*n* = 27761, mean age: 10.58 ± 4.53), 18,066 patients (65%) were male (mean age: 10.83 ± 4.46) and 9695 patients (35%) were female (mean age: 10.10 ± 4.61) (Table 1).

**Table 1:** Demographic data: sex and age distribution

	2000–2004 (abs. |rel.%)	2015–2019 (abs. |rel.%)	Total (abs. |rel.%)
Patients number	15,221	12,540	27,761
Male	9709 | 64%	8357 | 67%	18,066 | 65%
Female	5512 | 36%	4183 | 33%	9695 | 35%
Mean age in years (± SD)			
Total	11.17 (± 4.27)	9.85 (± 4.73)	10.58 (± 4.53)
Male	11.32 (± 4.24)	10.27 (± 4.65)	10.83 (± 4.46)
Female	10.91 (± 4.30)	9.03 (± 4.79)	10.10 (± 4.61)

The 2000–2004 subgroup consisted of 15,221 patients (54.8%) (mean age: 11.17 ± 4.27 years), of whom 9709 (64%) were male (mean age: 11.32 ± 4.24) and 5512 (36%) were female (mean age: 10.91 ± 4.30).

The 2015–2019 subgroup consisted of 12,540 patients (45.2%) (mean age: 9.85 ± 4.73 years), of whom 8357 (67%) were male (mean age: 10.27 ± 4.65) and 4183 (33%) were female (mean age: 9.03 ± 4.79).

The proportion of patients with leisure-related injuries among all injuries per period was significantly lower in the years 2015–2019 (total: 55971 patients) than compared to 2000–2004 (total: 46,172 patients) (22%; 12540/55971 vs. 33%; 15221/46173; *p* < 0.001).

### Epidemiological patterns by age groups

Stratified by age, the overall study population revealed the following: 4746 (17.1%) patients were up to 5 years of age, 6997 (25.2%) patients were between 6 and 10 years, 9911 (35.7%) patients were between 11 and 14 years and 6107 (22%) were between 15 and 17 years old (Table 2).

In both time periods 2000–2004 and 2015–2019, the majority of treated patients was between 11 and 14 years of age (38.8% vs. 31.9%; overall: 35.7%). However, the distribution of patients between each age group showed distinctive differences between studied time frames. In 2000–2004, 13% of patients were up to 5 years old, representing 41.6% of this particular age group. In 2015–2019, this number increased to 22,1% representing 58.4%. This change was deemed statistically significant (*p* < 0.0001).

In the two oldest age groups (11–14 and 15–17 years), a significant decline from the years 2000–2004 to 2015–2019 was observed (59.6% and 61.2% vs. 40.4% and 38.8%) (*p* < 0.0001; *p* < 0.0001). The 6–10 age group remained relatively stable.

**Table 2:** Study population stratified by age

Age	2000–2004 (abs. | column% | row%)	2015–2019 (abs. | column% | row%)	Total (abs. | column% | row%)
≤5 years	1975 | 13% | 41.6%	2771 | 22.1% | 58.4%	4746 | 17.1% | 100%
6–10 years	3595 | 23.6% | 51.4%	3402 | 27.1% | 48.6%	6997 | 25.2% | 100%
11–14 years	5911 | 38.8% | 59.6%	4000 | 31.9% | 40.4%	9911 | 35.7% | 100%
15–17 years	3740 | 24.6% | 61.2%	2367 | 18.9% | 38.8%	6107 | 22% | 100%
Total	15,221 | 100% | -	12,540 | 100% | -	27,761 | 100% | -

### Epidemiological patterns by anatomical region

**Fig. 2:**
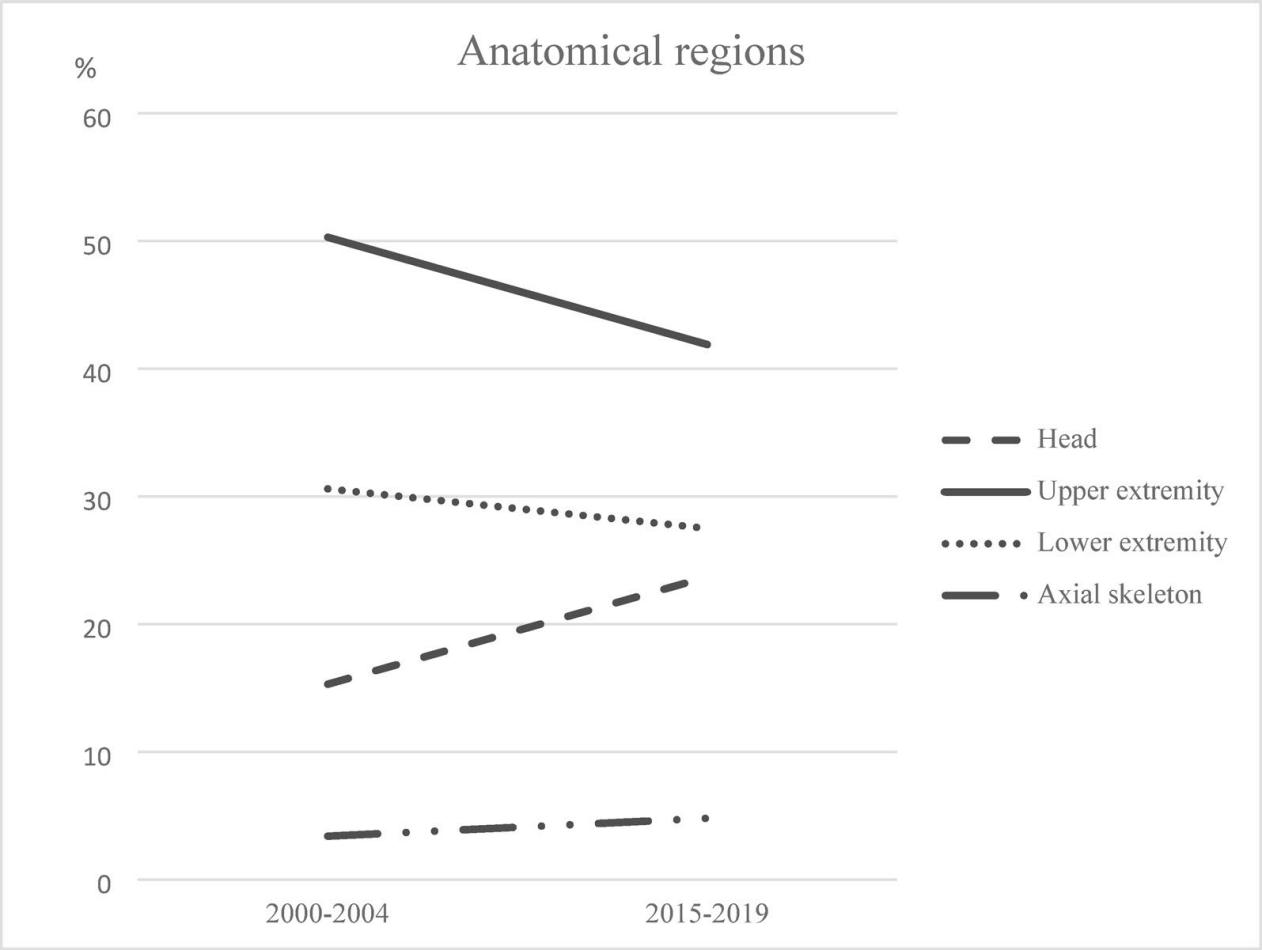
Trends of relative frequencies of affected anatomical regions between 2000–2004 and 2015–2019

Overall, the upper extremities were the most affected region in both groups with 50.3% in 2000–2004 and 41.9% in 2015–2019 followed by injuries of the lower extremities with 30.6% in 2000–2004 and 27.5% in 2015–2019 (Table 3). Notable was a rising trend of head injuries from 15.3% in 2000–2004 to 23.7% in 2015–2019 (Fig. 2).

**Table 3:** Anatomical region of injury

Anatomical region of injury	2000–2004 (abs. | rel. %)	2015–2019 (abs. | rel. %)	Total (abs. | rel. %)
Head	2334 | 15.3%	2977 | 23.7%	5311 | 19.1%
Upper extremity	7651 | 50.3%	5249 | 41.9%	12,900 | 46.5%
Lower extremity	4662 | 30.6%	3453 | 27.5%	8115 | 29.2%
Axial skeleton	514 | 3.4%	598 | 4.8%	1112 | 4%
Internal organs	11 | 0.1%	74 | 0.6%	85 | 0.3%
Not recorded	49 | 0.3%	189 | 1.5%	238 | 0.9%
Total	15,221 | 100%	12,540 | 100%	27,761 | 100%

### Head injuries

In total, 2295/5311 (43.2%) head injuries occurred in the up to 5 years age group. In this age group, a significant increase in head injuries was observed between the two periods (16.9% versus 31.5%; *p* < 0.0001). Head injuries in the other age groups remained relatively stable at between 5% and 10% in each group.

The most frequent head injury was found to be contusion with or without concomitant wounds (Table 4). The frequency of contusions without wounds raised between the two periods (44.8% in 2000–2004 vs. 49.9% in 2015–2019) whereas contusions with wounds showed a decline over time (43.1% in 2000–2004 vs. 40.4% in 2015–2019). Due to local hospital system, all patients were treated at our traumatological emergency department. The need for hospital admission was determined individually for most injuries except for concussion, fractures or intracranial hemorrhage. In these cases, all patients were admitted to standard care units for observation, routinely for 3–5 days (253 or 10.8% in 2000–2004 vs. 156 or 5.2% in 2015–2019). Of 3 patients with intracranial hemorrhage, 1 patient between 2000 and 2004 was admitted to ICU whereas the other 2 patients were admitted to standard care units.

Fall from a standing position was the most common mechanism of head injury (49.7% of the total study population; 50.2% in 2000–2004 vs. 49.4% in 2015–2019). Falls from heights greater than ½ meter were relatively rare (9.4% of the total study population). The next most commonly reported mechanisms were collisions with other people or objects and the utilization of a vehicle.

**Table 4:** Head injuries stratified by main diagnosis

Head injury	2000–2004 (abs. | rel.%)	2015–2019 (abs. | rel.%)	Total (abs. | rel.%)
Contusion	2051 | 87.9%	2689 | 90.3%	4740 | 89.3%
Concussion	156 | 6.7%	76 | 2.6%	232 | 4.4%
Fractures	95 | 4.1%	79 | 2.7%	174 | 3.3%
Intracranial hemorrhage	2 | 0.1%	1 | 0.03%	3 | 0.1%
Other (teeth, ears, nose etc.)	30 | 1.3%	132 | 4.4%	162 | 3.1%
Total	2334 | 100%	2977 | 100%	5311 | 100%

### Upper extremity

Overall, injuries to the upper extremities accounted for 46.5% of all injuries and were the most common injuries in all age groups except for the up to 5-year-olds. With 5561/12,900 (43.1%) of all upper extremity injuries, the most frequently affected patients were between 11 and 14 years old. Overall, a downward trend was observed in all age groups. The sharpest decline was recorded in the two adolescent groups (35.4% vs. 21.6% and 29.5% vs. 16.9%) (*p* < 0.0001; *p* < 0.0001).

### Lower extremity

Overall, almost one third of all injuries affected the lower extremities, making them the second most frequently affected region after the upper extremities. Once again, the age group most affected by these injuries was 11- to 14-year-olds (3033, 37.4%). In general, a decline was observed in all age groups, which was most pronounced in the two groups of adolescents (18.4% vs. 12.2%; 23.9% vs. 14.3%) (*p* < 0.0001; *p* < 0.0001).

### Axial skeleton and internal organs

Injuries to the axial skeleton and internal organs accounted for a small proportion of the study population in both periods and in all age groups. A total of 1112 injuries to the axial skeleton and 85 injuries to internal organs were documented. Injuries to the axial skeleton have hardly changed over time. Injuries to internal organs showed a slight upward trend.

### Epidemiological patterns by injury severity, type and mechanism

Most of observed injuries were considered mild in both time periods (67.4%, 10257/15221 vs. 73.3%, 9193/12540). Moderate injuries were per percentage 14% vs. 8.3%. The number of severe injuries remained relatively stable and was observed in 18% of cases in 2000–2004 and in 16.3% in 2015–2015 (Table 5). Fractures and wounds dropped from 10.3% to 10.8% in 2000–2004 to 6.3 and 6.4% in 2015–2019 (Table 6). Additionally, the most common mechanisms are illustrated in Table 7.

**Table 5:** Stratified by injury severity

Injury severity	2000–2004 (abs. | column% | row%)	2015–2019 (abs. | column% | row%)	Total (abs. | column% | row%)
Mild	10,257 | 67.4% | 52.7%	9193 | 73.3% | 47.3%	19,450 | 70.1% | 100%
Moderate	2128 | 14% | 67.2%	1039 | 8.3% | 32.8%	3167 | 11.4% | 100%
Severe	2735 | 18% | 57.2%	2044 | 16.3% | 42.8%	4779 | 17.2% | 100%
Not recorded	101 | 0.6% | 27.7%	264 | 2.1% | 72.3%	365 | 1.3% | 100%
Total	15,221 | 100% | -	12,540 | 100% | -	27,761 | 100% | -

**Table 6:** Trends of severe injuries (fractures and wounds)

	2000–2004 (*n* = 15221)	2015–2019 (*n* = 12540)	Total (*n* = 27761)
Severe injuries	(abs. | rel.%)	(abs. | rel.%)	(abs. | rel.%)
Fractures	2779 | 18.3%	2096 | 16.7%	4875 | 17.6%
Wounds	1643 | 10.8%	803 | 6.4%	2446 | 8.8%

**Table 7:** Most common injury mechanisms between both cohorts

Mechanism	2000–2004		2015–2019	
	*n*	%	*n*	%
Running	3769	24.8	503	4.0
Soccer	2369	15.6	2607	20.8
Playground	1122	7.4	2841	22.7
Winter sports	421	2.8	298	2.4
Handball	196	1.3	22	0.2
Skating	125	0.8	112	0.9
Trampoline	87	0.6	313	2.5

## Discussion

In this study, data of leisure time related injuries in children and adolescents over two decades showed a significant shift within affected anatomical regions. Injuries to the upper extremities remained most common but declined over time, as did lower extremity injuries. In contrast, head injuries showed a notable overall increase (from 15.3% to 23.7%) and particularly in the youngest age group (≤5 years) where the proportion of head injuries rose to 54% of all injuries in the latter period (an increase of almost 15%), which is consistent with the current literature [27]. Similar trends can be found in the US and in the UK: In the study population across England, Wales, Northern Ireland and the Channel Islands described by Trefan et al., children between 0 and 5 years of age made up for over 50% of recorded head injuries [28]. Nabavizadeh et al. reported a statistically significant increase in concussions over the course of 25 years between 1995 and 2019 [29]. This phenomenon can be attributed to a number of factors, including increased indoor activities, a lack of adequate supervision, parental anxieties, and easier access to healthcare facilities, resulting in a higher volume of visits to emergency departments [28, 30]. Other studies conducted in the US also showed increasing numbers of ED visits with the majority of patients being able to be discharged home [31, 32]. Contrary to this trend, higher hospitalization rates were reported in New Zealand, especially among 5–9-year-olds. Between 2012 and 2018, hospitalization rates increased 1.4% annually with a greater increase for female patients (0.5% vs. 2.4%), children between 5 and 9 years of age were 2.8 times more frequently admitted to hospital care [33].

The injury severity in this study was predominantly considered as mild in both time periods, with a slight increase over time. The proportion of moderate injuries declined, while severe injuries remained relatively stable. This development might suggest a generally safer environment or improved safety measures reducing the risk of serious injury [34]. Globally, the overall incidence of unintentional injuries as well as injury-related mortality among children and adolescents show decreasing trends [1, 32, 35]. However, the extent of this trend is influenced by a variety of factors including mechanisms of injury, demographic and cultural differences as well as local public health policies [1]. In the US, for example, an increase in firearm and cut injuries with a parallel decrease in blunt traumas and pedestrian injuries were reported [32].

Another observation of this study was a notable shift in age distribution. Again, the proportion of children of the age up to 5 years generally increasing in the latter period between 2015 and 2019 was highly noticeable. This observation may be linked to a currently discussed altered care-seeking behavior for minor injuries in toddlers [36–38]. Additionally, Poon et al. reported an increase in acute care for low-acuity cases between 2008 and 2015 [39]. In 2014, a referral system for ED visits was implemented in Denmark. Since then, decreased number of injuries were reported indicating a relation between ED access and injury incidence rates [40]. Further, similarly to Weimann et al. less injuries in older children and adolescents were observed during 2015–2019 [36]. A shift in injury risk due to environmental or behavioral changes could be a possible explanation [37]. The decreased number of older children may be further explained by enhanced safety measures in activities typically preferred by this age group [27].

However, the main observation of this study was that overall, significantly less children and adolescents were treated due to leisure and sports-related injuries in the years 2015–2019 compared to twenty years (2000–2004) ago. Räisänen et al. described a positive correlation between injury prevalence and the amount of physical activity [41]. This connection was later again supported by another European study [42]. These and the present findings could reflect changes in recreational behavior of children and adolescents and preferred leisure time activities as well as a decline in sport and activity levels in general. These assumptions could be coherent with the literature describing a decrease in cardiorespiratory endurance among children and adolescents while also reporting a smaller increase in muscle strength [38]. When it comes to sports or recreation-related injuries, different tendencies can be found around the world: while in New Zealand rising numbers of playground injuries were reported, the overall incidence in Scandinavian countries showed a downwards trend [27, 33, 43]. In the US, decreasing numbers of contact sports-related TBI and musculoskeletal injuries were reported [44, 45]. This observation of a significantly reduced frequency of injuries among children and young people during their leisure time may also be attributable to increased risk and safety awareness, an improved quality of safety and protective equipment, and new learning and training developments such as balance bikes for younger children before they actually learn to ride a bicycle [46]. Using the example of cycling, Thorsdottir et al. showed in their study the effect of safety measures and effective prevention strategies: the incidence rates for bicycle related injuries dropped by nearly 70% over the course of 40 years [47]. Furthermore, the observed shift towards fewer injured adolescent patients could be indicative of a preference for more concentrated physical activity in a gym setting, as opposed to active, engaging play. This hypothesis is supported by the findings of Loder and Johnson [48]. However, considering the usual popular recreational vehicles (bicycles) and the new upcoming trends such as scooters and e-scooters, some elevated numbers of injuries in this cohort may be expected also due to an increased availability and more frequent visiting of emergency departments. The hypothesis of reduced physical activity may also be further supported by the absence of legal obligations for children over the age of 12 to wear helmets while riding bicycles, scooters, or e-scooters at the local level which raises concerns regarding the efficacy of safety implementation measures. Under the assumption of steady activity levels among young people, this lack of legal implementation and the general aspect of increasing traffic would probably lead to an increase or at least a steady number of injuries. Furthermore, given the tendency of young people to generally receive less supervision from their parents during their free time would also allow for the conclusion of at least an even frequency of injuries. Considering all this, the authors’ assumption of a reduced number of leisure time related injuries indicating lower activity levels among children and adolescents appears to be valid and supported by the literature which describes a positive association between active play time and the risk of a necessary visit to the emergency room [46].

Limitations of this study include the retrospective design and possible inconsistencies in documentation practices between the chosen time frames. Inconsistent documentation denied a detailed analysis of the use of protective gear during certain activities. Although, the relationship between socioeconomic status and health related outcomes is well established in the literature [36, 49], such details are not routinely documented at our clinic and were therefore not part of this study. A potential limitation of the study is the age of the data, which is over six years old. Additionally, the increasing use of e-bikes and e-scooters by children during the years 2020–2026 might have led to a reverse trend. The data was reported until 2019, excluding the period of the pandemic. This period was intentionally omitted to avoid potential bias. On the one hand, by focusing on the pre-Covid era, authors sought to eliminate potential biases resulting from local regulations during the pandemic. Conversely, selecting a recent but shorter observation period for comparison would have resulted in disparate time periods, which would have introduced a significant bias. The increasing number of e-bikes and trendy e-scooters are relatively new and the numbers may not be that significant [25]. The substantial sample size of this study is both a key strength and a significant limitation. A manual data search is limited in terms of the resources available for determining precise risk factors and mechanisms under this retrospective design. Furthermore, data obtained from only a single center, however this clinic is considered one of the largest trauma centers worldwide represented by this large sample size and long observation period being unmatched in the literature. This representative large sample size enabled a detailed look at epidemiological changes, injury patterns and severity. On the one hand, these results underscore the importance of promoting physical activity, organized sports, and health awareness among children and adolescents. On the other hand, these results also highlight the importance of parental education, safety awareness and a possible extension of legal obligations to implement prevention strategies, such as helmet laws, particularly in relation to traffic-related activities.

### Conclusions

This study reveals notable shifts of leisure time associated injuries in the pediatric population in patient demographics, injury locations, and severity over two decades. Over the past twenty years, adolescents revealed significantly fewer injuries while children up to the age of 5 represent an increasingly vulnerable age group. This documented change requires age-appropriate measures to enhance physical activity among this specific population while ensuring a safe environment through the continued implementation of safety regulations.

## Data Availability

The datasets generated and/or analyzed in the current study are not publicly available due to data privacy but are available from the corresponding author on reasonable request.
